# Divergent cardiac and renal effects of miR-181c-5p inhibition in a rodent heart failure model

**DOI:** 10.3389/fcvm.2024.1383046

**Published:** 2024-04-25

**Authors:** Jente R. A. Boen, Andreas B. Gevaert, Amélie Dendooven, Dustin Krüger, Michiel Tubeeckx, Jens Van Fraeyenhove, Tine Bruyns, Vincent F. M. Segers, Emeline M. Van Craenenbroeck

**Affiliations:** ^1^ Research Group Cardiovascular Diseases, GENCOR Department, University of Antwerp, Wilrijk, Belgium; ^2^ Laboratory of Physiopharmacology, GENCOR Department, University of Antwerp, Wilrijk, Belgium; ^3^ Department of Cardiology, Antwerp University Hospital (UZA), Edegem, Belgium; ^4^ Department of Pathology, Ghent University Hospital, Gent, Belgium; ^5^ Laboratory of Experimental Medicine and Pediatrics, University of Antwerp, Wilrijk, Belgium

**Keywords:** heart failure, miR-181c-5p, renal dysfunction, thrombotic microangiopathy, fibrosis, diastolic dysfunction

## Abstract

**Aims:**

MiR-181c-5p overexpression associates with heart failure (HF) and cardiac damage, but the underlying pathophysiology remains unclear. This study investigated the effect of miR-181c-5p inhibition on cardiac function and fibrosis in a rodent model of diastolic dysfunction, and evaluated additional effects on kidney as relevant comorbid organ.

**Methods and results:**

Diastolic dysfunction was induced in male C57/BL6J mice (*n* = 20) by combining high-fat diet, L-NG-nitroarginine methyl ester, and angiotensin II administration, and was compared to sham controls (*n* = 18). Mice were randomized to subcutaneous miR-181c-5p antagomiR (INH) or scrambled antagomiR injections (40 mg/kg/week). HF mice demonstrated diastolic dysfunction and increased fibrosis, which was attenuated by INH treatment. Remarkably, HF + INH animals had a threefold higher mortality rate (60%) compared to HF controls (20%). Histological examination revealed increased glomerular damage in all INH treated mice, and signs of thrombotic microangiopathy (TMA) in mice who died prematurely. Quantitative polymerase chain reaction demonstrated a miR-181c-5p-related downregulation of cardiac but not renal *Tgfbr1* in HF + INH mice, while INH treatment reduced renal but not cardiac *Vegfa* expression in all mice.

**Conclusion:**

This study demonstrates cardiac anti-fibrotic effects of miR-181c-5p inhibition in a rodent HF model through targeting of *Tgfbr1* in the heart. Despite improved diastolic function, HF + INH mice had higher mortality due to increased predisposition for TMA, increased renal fibrosis and glomerular damage, associated with *Vegfa* downregulation in kidneys.

## 1 Introduction

Heart failure (HF) is a global health problem affecting 1%–3% of the adult population, with a 5-year mortality rate of 50%–75% (1–3). HF prevalence continues to rise due to an ageing population (1, 3–5), and leads to high hospitalization rates, high healthcare expenditures, and a low quality of life. It has been estimated that 50% of HF cases present with preserved ejection fraction (HFpEF; EF ≥ 50%), but a treatment that improves mortality is still not available for these patients (6, 7). HFpEF requires a personalized and highly organized care, including treatment of risk factors [hypertension, diabetes, hyperlipidemia, chronic kidney disease (CKD)] (7). Currently, the molecular mechanisms of HFpEF are incompletely understood. For instance, the driving mechanisms of left ventricular hypertrophy and myocardial fibrosis, and the links with non-cardiac comorbidities such as CKD remain largely undetermined (8, 9).

MicroRNAs (miRNAs) are small non-coding RNAs with a length of 18–24 nucleotides. They modulate cell–cell communication by transcriptional and post-transcriptional gene silencing/activation (10–13). MiRNA–target interactions depend on the abundance and accessibility of miRNAs and target mRNAs, timing, cell type and state (11, 14). MiRNAs control expression of over 60% of protein coding genes, highlighting their impact on cell physiology. Several miRNA-based therapies are under development in the field of cardiovascular disease (15). Specific miRNAs can potentially be used as biomarkers for noncommunicable diseases (16–18).

Recent studies demonstrate a role for miR-181c-5p in cardiovascular physiology, but its multi-organ crosstalk is understudied (19–23). In the heart, overexpression of miR-181c-5p is associated with enhanced propensity for HF development and cardiac damage (24–28). Increased levels of circulating miR-181c-5p are associated with diabetes-related HFpEF in elderly (29) and poor treatment response in HFpEF patients (30). Some studies suggest a cardioprotective effect of miR-181c-5p inhibition. Potential underlying mechanisms include improvement of endothelial and mitochondrial function, attenuation of myocardial fibrosis, and cardiorenal interaction, but these pathophysiologic mechanisms remain understudied. In this study we evaluate (1) the protective and anti-fibrotic role of systemic miR-181c-5p inhibition in HF progression using a rodent model with mild diastolic dysfunction, and (2) consider multi-organ effects at the level of the kidney as related comorbid organ in HF.

## 2 Materials and methods

### 2.1 Animals and experimental design

Diastolic dysfunction was induced in male C57/BL6J mice (Charles River Laboratories, *n* = 20) aged 15 weeks by combining three frequently used stimuli (31–33). For a period of six weeks animals were fed a 60% kcal high-fat diet (HFD, Research Diets Inc., D12492) together with L-N^G^-Nitro arginine methyl ester (L-NAME, Merck, N5751) administration in drinking water (0.5 g/L). In week five, angiotensin II (AngII, 1000 ng/kg/min, Merck; A9525) was co-administered for two weeks by osmotic minipumps (Alzet 1004, Charles River Laboratories) implanted subcutaneously under general anesthesia (4%–5% Sevoflurane). Control animals (*n* = 18) were fed normal diet and drinking water, and underwent sham-surgery at week five with PBS-filled vehicles. Two weeks after minipump implantation, invasive hemodynamic measurements were performed, and mice were euthanized at the age of 21 weeks by exsanguination.

**Figure 1:**
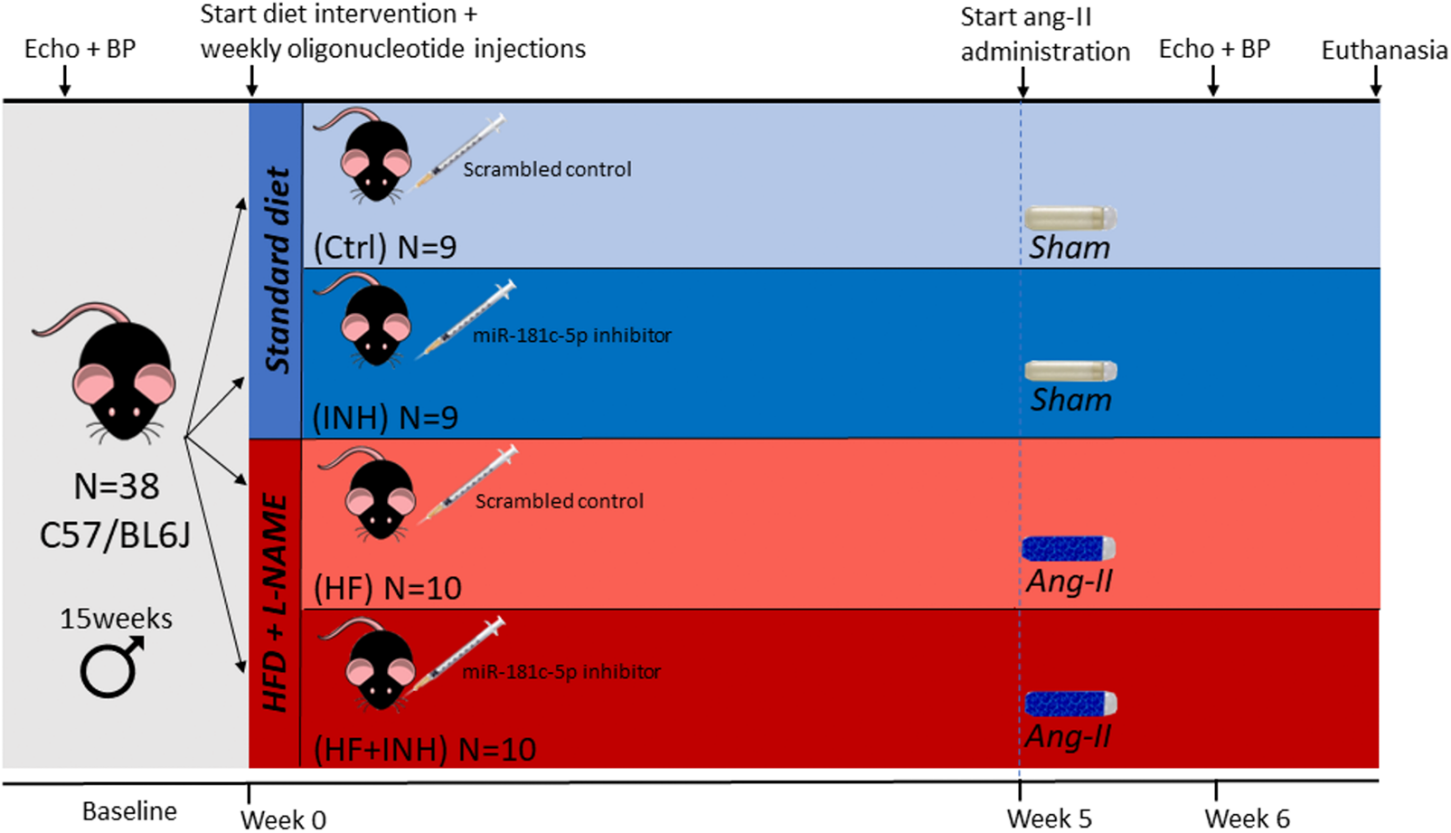
Experimental design of the study. The study consisted of four treatment groups: healthy controls with scrambled (Ctrl) or inhibitor injections (INH), and scrambled (HF) or inhibitor treated (HF + INH) animals with HF phenotype. We induced HF with diastolic dysfunction by administering NO synthase inhibitor L-NG-Nitro arginine methyl ester (L-NAME, 0.5 g/L) via drinking water and high fat diet (HFD, 60% fat) for six weeks, while angiotensin II (ang-II, 1,000 ng/kg/min) was co-administered in the final two weeks by osmotic minipumps. Healthy controls were given normal chow and drinking water, and sham-surgery was performed with PBS-vehicles. Subcutaneous injections (40 mg/kg) with either mmu-miR-181c-5p miRCURY power inhibitor or scrambled antagomiR occurred weekly for the duration of the study. After six weeks, we sacrificed the animals at the age of 21 weeks. BP, blood pressure.

All animals were housed in a facility with 12 h dark-light cycle and both food and water *ad libitum* (Figure 1). Animals that died prematurely without completion of all measurements (*n* = 7) were not included for analyses. 2 animals (INH group) were excluded because of fighting injuries during the study. All animal experiments were performed according to the latest *Guide for the Care and Use of Laboratory Animals* (National Institute of Health) and were approved by the Ethical Committee for Animal Testing at University of Antwerp (LA1100154), and have therefore been performed in accordance with the ethical standards laid down in the 1964 Declaration of Helsinki and its later amendments.

### 2.2 Oligonucleotide administration

Both mice assigned to the HFD/l-NAME and the standard diet group were at baseline randomly allocated to either weekly injections with mmu-miR-181c-5p miRCURY power inhibitor (Qiagen, 339203, YCI0201911-FZA) or scrambled antagomiR (Qiagen, 339204, YCI0202244-FZA) (Supplementary Table S1). Injections were performed subcutaneously once weekly for the duration of the study at a dose of 40 mg/kg (Figure 1). This dosage was determined in previous dose response experiments, which also demonstrate inhibitor efficiency (Supplementary Figure S1). The study consisted of four treatment groups: (1) Ctrl: healthy controls with scrambled antagomiR injections; (2) INH: healthy controls with miR-181c-5p inhibitor injections; (3) HF: HF animals with scrambled antagomiR injections; (4) HF + INH: HF animals with miR-181c-5p inhibitor injections.

### 2.3 Blood pressure measurements

Blood pressure was measured in conscious mice at baseline and at week six with a CODA electronic sphygmomanometer, using a tail-cuff system (Kent Scientific Corporation). For each mouse, five acclimatization cycles followed by 20 consecutive blood pressure measurements were obtained. Data were extracted with CODA v4.1 Software. For each animal, the average of at least eight successful measurements was required.

### 2.4 Echocardiography

Systolic and diastolic function were analyzed with cardiac ultrasound using a Vevo 2100 Ultrasound imaging system (VisualSonics) with 55-MHz transducer (MS550D). Mice were held under light anesthesia [1.5%–2.5% v/v isoflurane (Forene Abbvie)] during imaging, temperature was held constant at 37 ± 1°C by a heating pad. For examination of left ventricular (LV) diameters the transducer was placed in a parasternal long axis position. Measurement was performed in M-mode, with the cursor position set at the level of the papillary muscles. Ejection fraction, LV mass and fractional shortening were calculated from these parameters. Left atrial (LA) diameter was measured near the aortic valve. Diastolic function was evaluated via apical four-chamber view using pulse wave doppler and tissue doppler imaging. Pulse wave doppler signals were obtained at the level of the LV inflow, tissue doppler measurements were obtained at the septal mitral annulus. E/e’ and E/A were derived from these parameters. Three images were taken from every parameter per animal and analyzed with Vevo Lab software (Version 3.2.0, VisualSonics) by a blinded observer.

### 2.5 Invasive hemodynamics

Mice were anesthetized using sevoflurane at concentrations of 8% v/v for induction and 4%–5% v/v for maintenance. The animals were placed on a heating pad (Kent Scientific) to maintain body temperature at approximately 37°C while free breathing. Throughout the experimental procedure, warm saline at 37°C was regularly applied to the open wound to prevent fluid loss. A pressure-volume catheter (Millar SPR-839) was inserted into the left ventricle through the right carotid artery using a closed-chest approach. To calculate parallel conductance (or parallel volume, Vp) and estimate the absolute ventricular volume, a catheter connected to a syringe containing hypertonic saline (15% NaCl) was introduced and secured in the right jugular vein, and a 10 µl-bolus injection of hypertonic saline was repeated three times. The end-diastolic pressure-volume relationship (EDPVR) was obtained through preload reduction induced by occluding the inferior vena cava, and fitting an exponential curve to end-diastolic pressure-volume points (LabChart v8, AD Instruments).

### 2.6 Organ weight

Organs were collected for histological and molecular analyses. Both cardiac mass and kidney mass were determined, as well as tibia length. Lung mass was weighted in wet and dry condition before and after a drying period of 72 h in an incubator at 37°C to evaluate lung congestion.

### 2.7 Histology

Frontal sections of renal and cardiac tissue were fixated in buffered formol for 24 h followed by a dehydration step in 60% isopropanol and embedding in paraffin. Sections of 5 µm were deparaffinized, rehydrated and treated with 0.9% hydrogen peroxide for blocking endogenous peroxidase activity. After antigen retrieval, permeabilization with TSB-tritonX and blocking with diluted serum solution (1/10), primary antibodies were incubated overnight at 4°C. Secondary antibodies were added for 30 min at room temperature after several washing steps. In case of HRP-labeled antibodies, an additional step with 3,3′-diaminobenzidine was required.

Slides were imaged with an Olympus BX43 light microscope equipped with Olympus Stream Motion software or Hamamatsu C10730-12 scanner microscope with NDP2 view software, while for isolectin B4 staining a Nikon Eclipse T1 fluorescence microscope was used with NIS advanced research software. All images were analyzed with ImageJ v1.53, and semiquantitative histological analyses were performed by two observers, all in blinded fashion.

#### 2.7.1 Masson’s trichrome staining

Masson's Trichrome staining was used to visualize renal and cardiac interstitial and perivascular fibrosis. For perivascular fibrosis, 10 blood vessels per animal were randomly analyzed at 20X magnification. For interstitial fibrosis, 6 random images for each animal were analyzed at 20X magnification.

#### 2.7.2 Hematoxylin eosin (H&E) staining and periodic acid-shiff staining (PAS)

H&E and PAS staining were used to evaluate renal morphology. For glomerular investigation, 50 glomeruli on two independent sections of each animal were randomly studied for glomerular size and for the presence of global/segmental glomerulosclerosis and mesangial matrix expansion. The average glomerular size and percentage of damaged glomeruli was calculated.

#### 2.7.3 WT-1 staining

WT-1 staining (Abcam, ab224806) was used to determine podocyte density evaluated by counting the number of podocytes per glomerular area. 25 glomeruli were randomly analyzed for each animal at 20X magnification.

#### 2.7.4 Cd31 staining

CD31 staining (Cell Signaling, 77699) was used to determine endothelial density in global and glomerular renal tissue. 25 glomeruli on two independent images were randomly analyzed for each animal.

#### 2.7.5 Isolectin B4 staining

Isolectin B4 staining (Invitrogen, I21411) was used to determine capillary density in the heart. The number of capillaries was counted from five independent images for each animal at 40X magnification.

### 2.8 Reverse transcriptase quantitative polymerase chain reaction

The expression of renal and cardiac mRNA of potential target genes (*Tgfbr1, Vegfa, Smad7*) (Supplementary Table S2) and the expression level of miR-181c-5p was quantified with multiplex reverse transcriptase quantitative polymerase chain reaction (RT-qPCR). Potential targets were determined based on prediction programs, pathway analysis and current literature. Total RNA was homogenized with Precellys Tissue homogenizer (Bertin Instruments) and extracted from snapfrozen cardiac or renal tissue (10–20 mg) with mirVana™ PARIS™ RNA and Native Protein Purification Kit (Invitrogen, AM1556) according to manufacturer guidelines. RNA quality was controlled with Nanodrop ND-1000 spectrophotometer (Thermofisher Scientific). For mRNA targets, cDNA synthesis was performed with the Reverse transcription reagent kit (Applied Biosystems, N8080234) and subsequently used for qPCR using Taqman Universal master mix no UNG (Applied Biosystems, 4440047) and FAM-labeled TaqMan primers (Thermofisher Scientific). For miRNA levels, reverse transcription step started with cDNA-synthesis using TaqMan MicroRNA Reverse Transcription Kit (Thermofisher Scientific, 4366596), followed by a preamplification step with TaqMan™ PreAmp Master Mix (Thermofisher Scientific, 4488593). The qPCR step was performed as described above. Relative expression was analyzed with 2^(-ΔΔcq) method, using *Gapdh* and β-*actin* for renal and *Ywhaz* and *Rplpo* for cardiac mRNA reference genes (Supplementary Table S3). For miR-181c-5p expression, *Sno142* and *Sno202* were applied as normalization genes (Supplementary Table S4). No-reverse transcriptase control and no-template controls were used to assess sample or product contamination.

### 2.9 Statistics

Data were analyzed with GraphPad Prism 9.0. Graphs are represented with mean ± SD or in case of non-Gaussian distribution with median ± IQR. For comparison of two groups, a nonpaired two-tailed Student's *T*-test was performed. For statistical testing in multiple groups with several independent between-factors (i.e., treatment and phenotype) with or without a within-factor (i.e., time), a two-way ANOVA or mixed model ANOVA with Tukey's test or Šidák test for multiple comparisons was performed. Survival rate was analyzed with Kaplan Meier using a log-rank test. For end diastolic pressure-volume (EDPVR) curves, non-linear regression with least squares fit was performed. Alpha level was set at 0.05. Normality of data was tested with Shapiro-Wilk test. In case of skewed data, nonparametric testing or log-transformation was performed where possible.

## 3 Results

### 3.1 MiR-181c-5p inhibition mildly attenuated diastolic dysfunction

**Figure 2:**
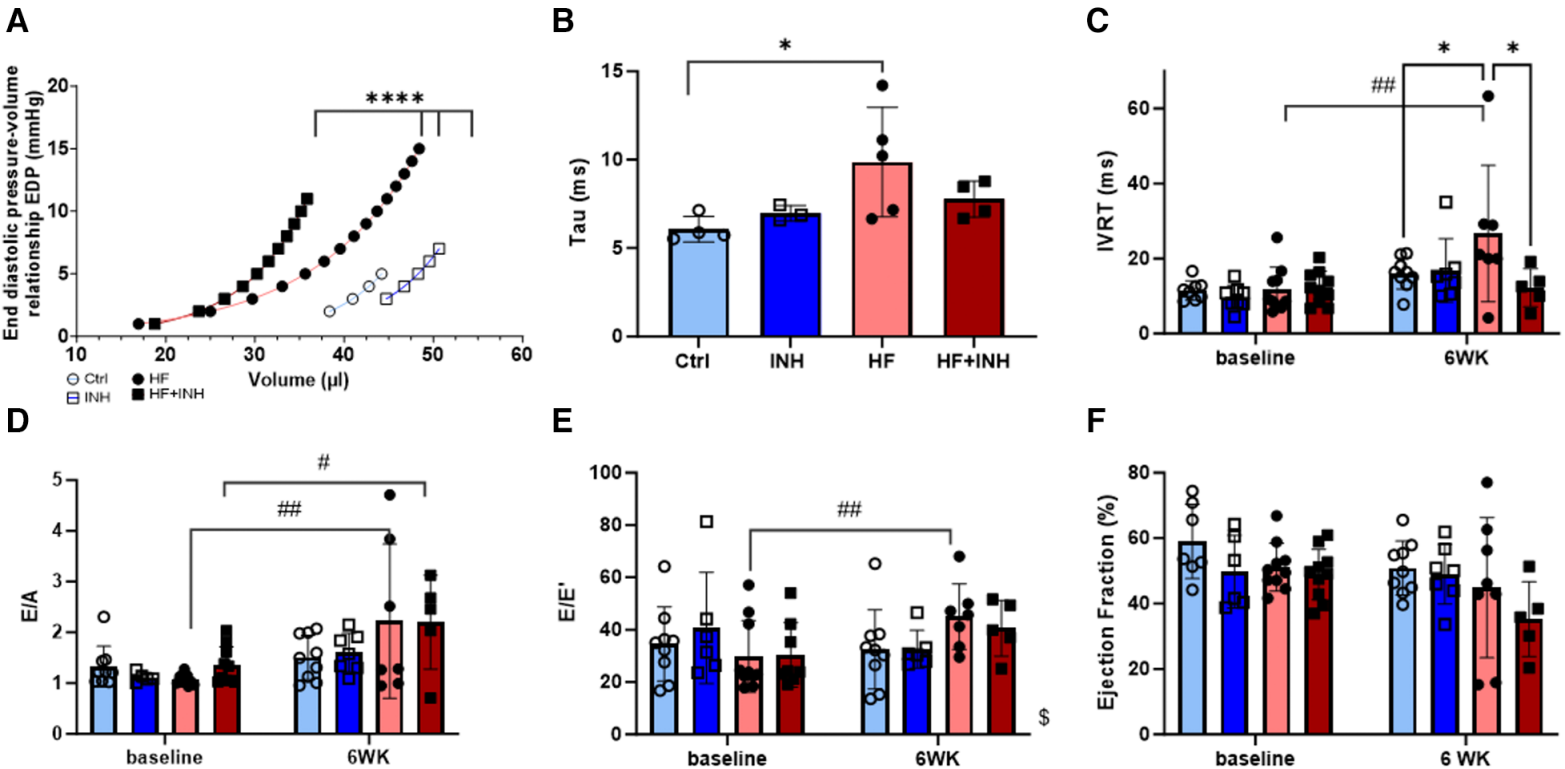
Cardiac function determined by invasive hemodynamics and echocardiography. Invasive hemodynamics validate diastolic dysfunction in HF mice with (**A**) leftwards shift of end diastolic pressure volume curve, and (**B**) increase of relaxation constant tau. Echocardiographic measures of diastolic dysfunction in HF mice illustrate an increase of (**C**) isovolumic relaxation time (IVRT), (**D**) E/A ratio over a period of six weeks— E/A represents the early (**E**) and late (**A**) ventricular filling velocities— and (**E**) E/e’ ratio over a period of six weeks — E/e’ represents the early mitral inflow over early mitral annular diastolic velocity. (**F**) Ejection fraction. *N* = 3-5 per group for invasive hemodynamics, *N* = 5-10 per group for echocardiography. Mixed-effect model for repeated measurements over time with Tukey's test or Šidák test for multiple comparisons. Two-way ANOVA with Šidák test for multiple corrections for single measurements. Non-linear regression with least square fit for edpvr curves. Mean ± SD, *p < 0.05, ****p < 0.0001, #*p* < 0.05 with baseline, ##*p* < 0.002 with baseline, $*p* < 0.05 interaction time × treatment. WK, week; IVRT, isovolumic relaxation time; EDP, end diastolic pressure; LV, left ventricular.

HF mice showed impaired diastolic function, partly attenuated by inhibitor treatment (Figure 2).

EDPVR curves demonstrate increased LV stiffness in HF mice represented by a leftward shift of the curve (Ctrl vs. HF *p* < 0.001). Inhibitor treatment in HF mice partially prevented this shift (*p* < 0.001), indicating a lower grade of LV stiffness. Similarly, HF mice show higher values for isovolumic relaxation constant tau (Ctrl 6.0 ± 0.6 ms, HF 9.3 ± 3.1 ms, *p* = 0.03), which are slightly reduced in HF + INH mice (HF + INH 7.8 ± 1.0 ms, *p* = 0.4 vs. HF). Echocardiography demonstrated a prolonged isovolumic relaxation time (IVRT) in HF mice (Ctrl 16 ± 4 ms, HF 27 ± 18 ms, *p* = 0.04), while inhibitor treatment normalized these values (HF + INH 14 ± 7 ms, *p* = 0.03 vs. HF). E/A and E/e’ did not differ between HF group and HF + INH group (E/A: HF 2.2 ± 1.5, HF + INH 2.2 ± 0.9, *p* > 0.99; E/e’: HF 45 ± 13, HF + INH 41 ± 11, *p* = 0.7). Ejection fraction was not significantly altered between groups over time, though a numerical drop was seen in the HF + INH group (42 ± 10%). None of the groups had evidence of pulmonary congestion.

### 3.2 MiR-181c-5p inhibition partly attenuated cardiac remodeling

**Figure 3:**
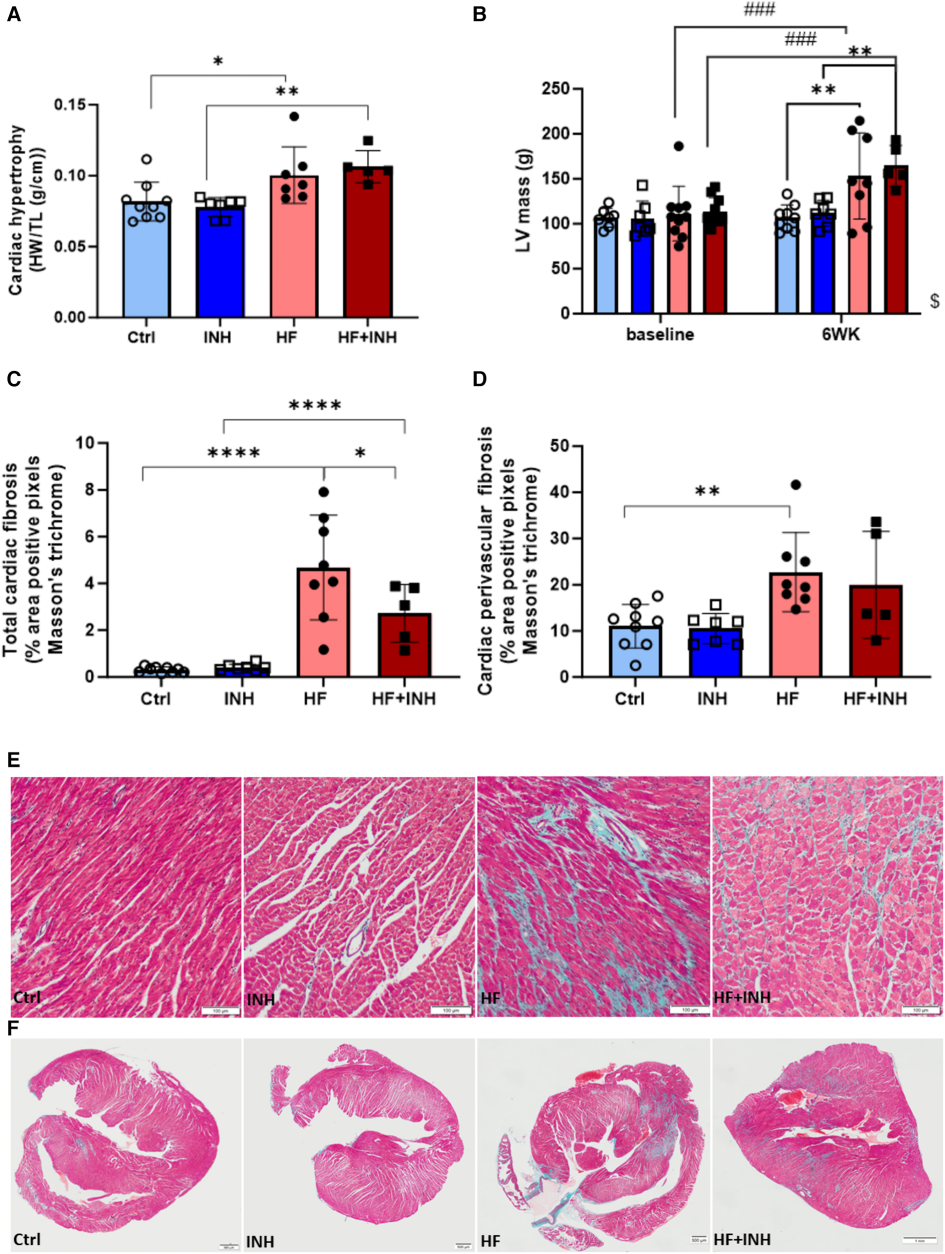
Cardiac morphology. (**A**) Heart weight over tibia length. (**B**) Left ventricular mass validated by echocardiography, (**C–F**) Total cardiac fibrosis and perivascular fibrosis determined by average percentage positive pixels with Masson's trichrome staining. *N* = 5-10 per group, 2-way ANOVA, mean ± SD. Mixed-effect model for repeated measurements over time with Tukey's test or Šidák test for multiple comparisons.*p < 0.05, **p < 0.002, ****p < 0.0001, ###*p* < 0.0002 with baseline, $*p* < 0.05 interaction time × treatment. HW, heart weight; TL, tibia length; LV, left ventricular; WK, week.

HF mice displayed altered cardiac structure, which was partly attenuated by miR-181c-5p inhibition (Figure 3).

HF mice had an increased heart weight (Ctrl 82 ± 14 mg/mm, HF 100 ± 20 mg/mm, *p* = 0.03), which was confirmed by echocardiography (LV mass: Ctrl 107 ± 14 mg, HF 153 ± 48 mg, *p* = 0.002). Inhibitor administration did not prevent the development of cardiac hypertrophy (heart weight: HF + INH 106 ± 11 mg/mm, *p* = 0.7 vs. HF; LV mass: HF + INH 165 ± 23 mg, *p* = 0.9 vs. HF).

Histological analysis demonstrated increased cardiac fibrosis in HF mice (Ctrl 0.31 ± 0.12%, HF 4.96 ± 1.84%, *p* < 0.0001), which was significantly reduced by inhibitor treatment (HF + INH 2.72 ± 1.24%, *p* = 0.02 vs. HF). Looking at the location of fibrosis, increased perivascular fibrosis was seen in HF mice compared to healthy mice (Ctrl 11.02 ± 4.70% vs. HF 22.748 ± 8.55%, *p* = 0.005). However, perivascular fibrosis was not attenuated by inhibitor injection in HF animals (19.97 ± 11.56%, *p* = 0.7 vs. HF). Capillary density was similar between groups.

### 3.3 MiR-181c-5p inhibition induced additional renal damage in HF mice

**Figure 4:**
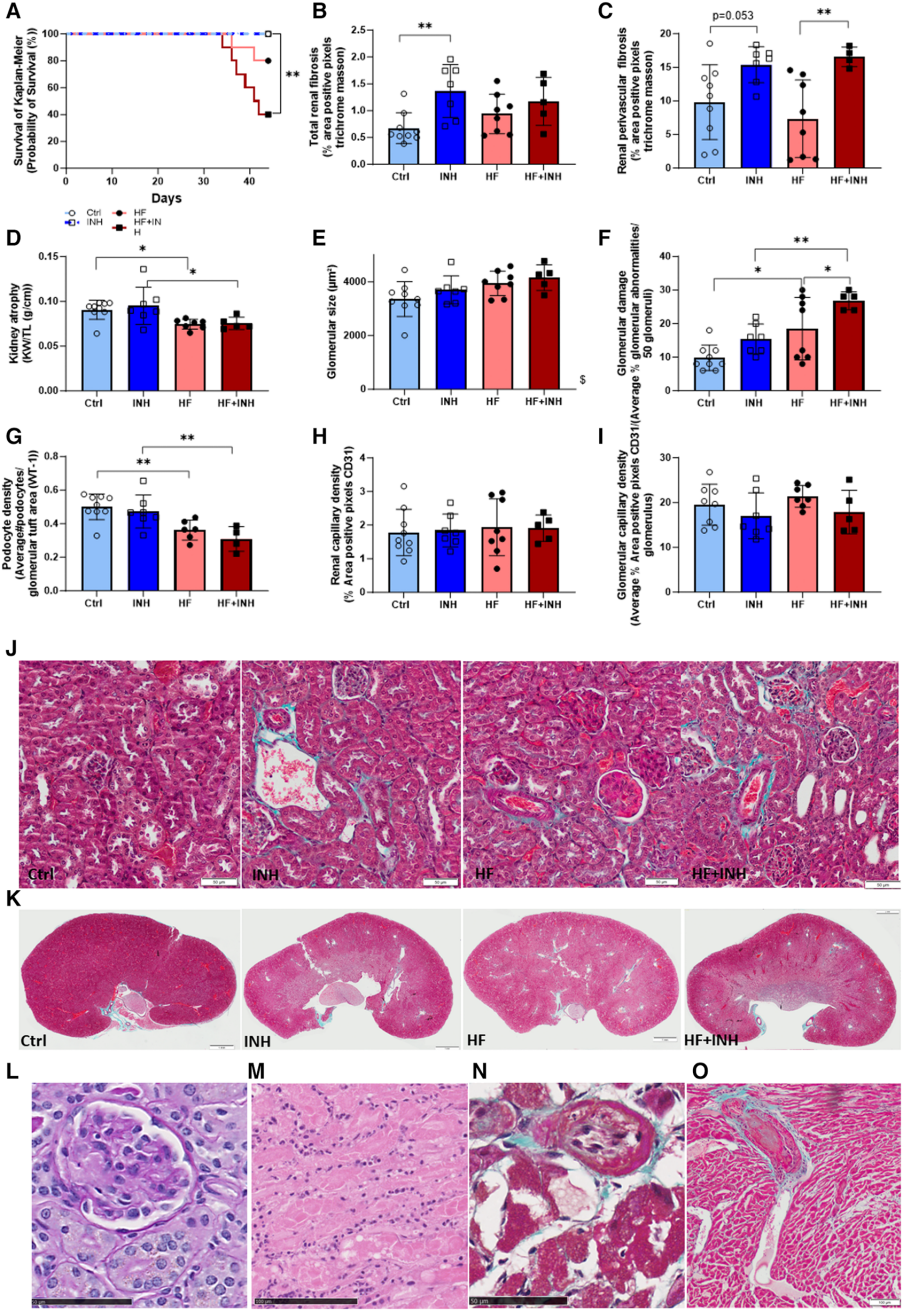
Renal morphology and thrombotic microangiopathy (TMA)-associated phenotype. (**A**) Survival analysis with Kaplan-Meier, (**B,C,J,K**) total and perivascular renal fibrosis determined by average percentage positive pixels with Masson's trichrome staining. (**D**) Kidney weight. Glomerular structure evaluated by (**E**) glomerular size determined on PAS staining, (**F**) glomerular damage determined on PAS staining, (**G**) podocyte density determined with WT-1 staining. (**H,I**) Renal and glomerular capillary density evaluated on CD31 staining. Premature deaths showed characteristic features of TMA in renal tissue, with (**L**) glomerular endothelial swelling and fibrine clots (*) on PAS staining, (**M**) tubular necrosis with inflammatory cells on H&E, while also extended to cardiac tissue showing (**N**) intimal thickening of cardiac artery and (**O**) thrombus formation (*) in necrotic region with inflammatory cells on Masson's trichrome staining. *N* = 5-9 per group. Kaplan Meier analysis with log-rank test. Two-way ANOVA with Šidák test for multiple comparisons. Mean ± SD, *p < 0.05, **p < 0.002, $*p* < 0.05 interaction time × treatment. KW, kidney weight; TL, tibia length.

HF mice had signs of renal damage (Figure 4), and inhibitor treatment induced additional renal damage in both healthy and HF mice.

HF mice had a reduced kidney weight compared to healthy mice, which was not affected by inhibitor administration (Ctrl 91 ± 11 mg/mm, HF 74 ± 5 mg/mm, *p* = 0.04; INH 95 ± 21 mg/mm, HF + INH 75 ± 7 mg/mm, *p* = 0.03). Likewise, podocyte density (which drives glomerulosclerosis and CKD) was lower in HF mice compared to healthy counterparts (Ctrl 0.501 ± 0.077/µm^2^, HF 0.362 ± 0.060/µm^2^, *p* = 0.007), but without any major effects of inhibitor treatment.

Renal interstitial and perivascular fibrosis was similar between Ctrl and HF mice (interstitial fibrosis: Ctrl 0.68 ± 0.29%, HF 0.94 ± 0.37%, *p* = 0.33; perivascular fibrosis: Ctrl 9.80 ± 5.56%, HF 7.35 ± 5.76%, *p* = 0.5). However, interstitial fibrosis significantly increased by inhibitor treatment in healthy mice, and to a lesser extent in HF conditions (interstitial: INH 1.37 ± 0.49%, *p* = 0.0036 vs. Ctrl; HF + INH 1.17 ± 0.45%, *p* = 0.5 vs. HF). We found large areas of perivascular fibrosis in all inhibitor-treated groups (INH 15.38 ± 2.70%, *p* = 0.053 vs. Ctrl; HF + INH 16.56 ± 1.46%, *p* = 0.008 vs. HF).

Histological H&E staining and PAS staining illustrated glomerular damage in HF animals, which was more pronounced in HF + INH group. HF animals had an enlarged glomerular size (*p* = 0.019) compared to healthy littermates, which is an indicator of glomerular hyperfiltration. HF mice also showed a higher percentage of additional glomerular damage— segmental and global glomerulosclerosis, mesangial matrix expansion and onset phase of sclerosis —(Ctrl 9.78 ± 3.80%, HF 18.50 ± 3.90%, *p* = 0.01; INH 15.43 ± 4.43%, HF + INH 26.80 ± 2.68%, *p* = 0.006), which was significantly increased when miR-181c-5p was inhibited (HF vs. HF + INH *p* = 0.04). We did not observe any significant differences in renal or glomerular capillary density.

### 3.4 MiR-181c-5p inhibition increased mortality through renal damage

HF mice had a higher mortality rate when administered with miR-181c-5p inhibitor (Figure 4, Supplementary Figure S2), likely due to a higher predisposition to thrombotic microangiopathy (TMA).

Mortality was 60% in the HF + INH group, 20% in the HF group, and 0% in both control and INH groups (log-rank *p* = 0.003, Figure 4A). All mice with premature death showed a phenotype compatible with TMA: tubular damage (acute tubular necrosis, tubular dilation) and glomerulomegaly, often combined with microvascular thrombi in glomerular capillaries and endothelial swelling. Fibrin clots were also detected in cardiac vessels of mice with renal TMA, and coexisted with necrotic regions and intimal thickening in the heart. At the level of the abdominal aorta, blood clots were detected during dissection in two HF + INH mice. Additionally, these mice demonstrated some degree of chronic renal damage under the form of glomerulosclerosis, interstitial fibrosis and tubular necrosis.

### 3.5 *Tgfbr1* and *vegfa* as potential targets of miR-181c-5p

**Figure 5:**
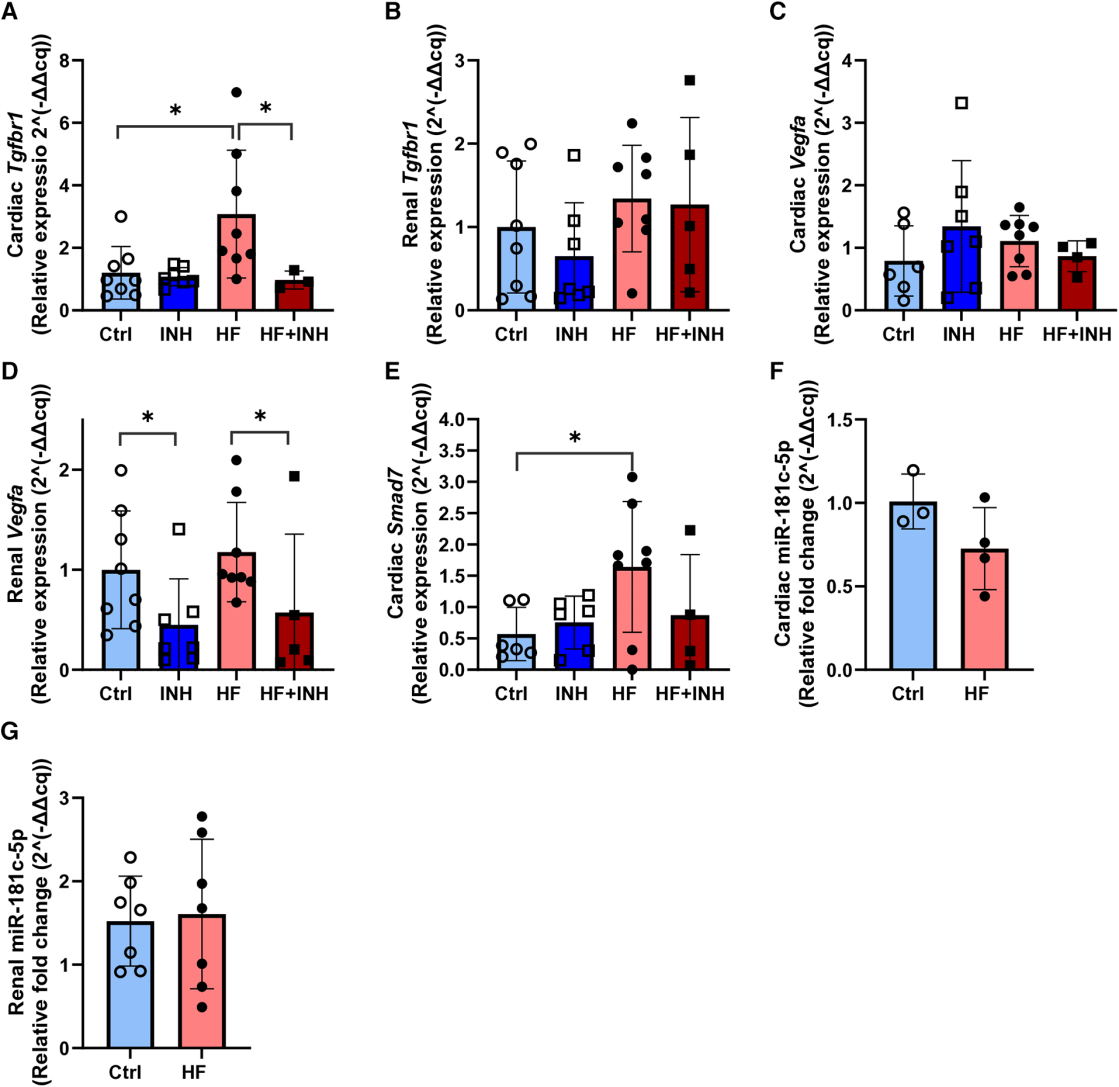
Expression of mRNA targets and miR-181c-5p in cardiac and renal tissue. (**A,B**) Cardiac and renal target expression of *Tgfbr1*. (**C,D**) Cardiac and renal target expression of *Vegfa*. (**E**) Cardiac expression of *Smad7.* (**F,G**) Cardiac and renal expression of miR-181c-5p. *N* = 3-8 per group. Evaluated with RT-qPCR and relative quantification according to 2^(-delta delta cq) method with two-way ANOVA followed by Tukey's test for multiple comparisons or unpaired *t*-test for miR-181c-5p. Mean ± SD, *p < 0.05.

HF mice showed increased expression of cardiac *Tgfbr1* and renal *Vegfa* (Figure 5), which was reduced by inhibitor treatment.

In the heart, we detected higher expression levels of *Tgfbr1* mRNA in HF mice compared to healthy animals (Ctrl 1.2 ± 0.8, HF 3.1 ± 2.0, *p* = 0.01). However, inhibition of miR-181c-5p normalized *Tgfbr1* expression in the HF + INH group (HF + INH 0.97 ± 0.29, *p* = 0.04 vs. HF), which confirms that *Tgfbr1* is a direct/indirect target of miR-181c-5p in the heart. Expression of cardiac *Vegfa* mRNA did not differ between groups. *Smad7* mRNA was increased in the HF group (Ctrl 0.46 ± 0.37, HF 1.64 ± 1.04, *p* = 0.03), and numerically lowered in HF + INH, but without significant effect of inhibitor treatment (HF + INH 0.89 ± 0.97, *p* = 0.24 vs HF).

In contrast, *Vegfa* mRNA levels in renal tissue were halved in inhibitor-treated groups compared to scrambled groups, regardless of their phenotype (Ctrl 1 ± 0.59, INH 0.45 ± 0.46, *p* = 0.01; HF 1.18 ± 0.50, HF + INH 0.57 ± 0.78, *p* = 0.04). This demonstrates a miR-181c-5p-mediated regulation of *Vegfa* in kidney*.* Renal expression of *Tgfbr1* was not influenced by treatment or phenotype.

### 3.6 Model-related effects on miR-181c-5p expression

The HF model did not influence cardiac and renal expression of miR-181c-5p (Figure 5).

Cardiac and renal expression of miR-181c-5p was analyzed in animals injected with scrambled antagomiR. MiR-181c-5p expression in cardiac tissue was slightly lower in HF mice, but without reaching statistical significance (Ctrl 1.01 ± 0.16, HF 0.73 ± 0.25, *p* = 0.14). Similarly, the HF phenotype did not affect renal expression of miR-181c-5p (Ctrl 1.52 ± 0.54, HF 1.61 ± 0.90, *p* = 0.83).

## 4 Discussion

We found that systemic inhibition of miR-181c-5p attenuated the development of myocardial fibrosis in a rodent model of mild diastolic dysfunction, in part by modulating *Tgfbr1*-signaling. However, the threefold higher mortality rate opposes these beneficial cardiac effects, and shifts the perspective from cardiac hero to renal villain: systemic miR-181c-5p inhibition caused renal damage (fibrosis, glomerular damage)—even in healthy mice— and predisposed to TMA development. These unexpected findings were explained by a miR-181c-5p-mediated reduction in renal *Vegfa* expression.

Multiple studies have shown that miR-181c-5p overexpression mostly harms the heart, while miR-181c-5p downregulation has cardioprotective effects (24, 25, 27, 34). Potential mechanisms by which increased miR-181c-5p affects cardiac structure and function include mitochondrial dysfunction, ROS production, inflammation, apoptosis and endothelial dysfunction (24–27, 34). A few studies have already proven the clinical value of miR-181c-5p: increased levels of circulating miR-181c-5p were detected in aged HFpEF patients with diabetes (29) and in HFpEF patients with a low response for exercise training (30). Recent *in vitro* studies in human cardiac fibroblasts identified SMAD7 as a new cardiac target of miR-181c-5p, which suggests a potential role for miR-181c-5p in cardiac fibrosis (29). In this study we now confirm *in vivo* an effect of miR-181c-5p on cardiac fibrosis through the TGF-β axis.

SMAD7, an inhibitor of TGF-β-signaling, was identified by the research group of Jankauskas et al. as a target of miR-181c-5p in cardiac fibroblasts, which we confirmed during our *in vivo* dose-finding studies (Supplementary Figure S1) in healthy cardiac tissue. While we expected a synergetic effect of miR-181c-5p inhibition on cardiac fibrosis by targeting both *Tgfbr1* and *Smad7* in HF conditions, we found that cardiac *Smad7* did not significantly differed from the HF scrambled group, and was rather downregulated instead of upregulated as seen in healthy mice. The exact explanation remains unclear, but we assume that the complex miRNA target dynamics (11) —which are sensitive towards internal and external triggers, like disease conditions or stress— might underly our findings. Examination of multiple disease models at different time points and further pathway analysis could provide more insights into the miR-181c-5p/*Smad7* axis. However, based on the expression results in this study, most anti-fibrotic effects probably associate with *Tgfbr1*-targeting. Therefore they clearly show a modulatory role for miR-181c-5p in Tgf-ß-signaling.

Our cardiac findings align with a study that reported reduced cardiac fibrosis after systemic miR-181c-5p inhibition in a mouse model with coronary artery disease through SIRT1-targeting (34). Interestingly, SIRT1 can inhibit *Tgfbr1* levels and affect fibrosis-related TGF-β-signaling (35). We assume that this pathway might substantiate our findings; we identified an atypical downregulation of cardiac *Tgfbr1* mRNA upon miR-181c-5p inhibition, which might refer to indirect targeting. However, the exact mechanism remains unclear; the research group of He X et al. identified TGFBR1 as a direct target of miR-181c-5p with luciferase reporter assay in glioblastoma cells (36). More research is required to unravel the exact *Tgfbr1*-interaction in cardiac tissue.

The effects of cardiomyocyte-specific *Tgfbr1* knockdown have been studied in a murine model (37) and correspond to our study outcomes: cardiac alterations were restricted to cardiac fibrosis, with minimal impact on cardiac hypertrophy or function. This is in contrast to observations of Roman et al, who described antihypertrophic effects of transgenic miR-181c/d deletion in a murine model of obesity (24). We expect that differences in timing of miR-181c modulation, incomplete suppression of miR-181c by antagomiR, or supplementary effects of miR-181d might have led to this discrepancy (38).

The ability of microRNAs to target multiple mRNAs in a tissue-dependent manner challenges current research and requires precaution for data interpretation (39). However, most studies only focus on the organ of interest. A multi-organ approach can provide more insights about ongoing events in comorbid organs, certainly in case of systemic miRNA interventions. In this study we observed severe renal damage in mice treated with miR-181c-5p inhibitor, in both healthy and HF conditions, which can influence heart-kidney crosstalk. As a result, these multi-organ effects can bias our understanding of miR-181c-5p in the isolated heart and underestimate the effect on cardiac function. A targeted approach for cardiac-specific delivery of miRNAs could eliminate the influence of these multi-organ effects (40).

In kidney, one paper reported a protective effect of miR-181c mimics against cyclosporine-associated renal toxicity *in vitro* and *in vivo* by attenuating renal fibrosis and improving renal function (41). Wenjuan et al. illustrated that ectopic miR-181c administration suppressed the immune response in sepsis-induced acute kidney injury (42). Our results confirm these deleterious renal effects of low miR-181c-5p levels, and they demonstrate for the first time the adverse renal alterations, particularly interstitial fibrosis, in healthy mice. We demonstrate that these outcomes partially relate to miR-181c-5p-mediated effect on *Vegfa*-signaling. However, it is likely that other unrecognized pathways participate (like inflammation, ROS) (41, 42).

An earlier study postulated that miR-181c-5p indirectly targets *Vegfa* via HIF1ɑ in endothelial cells validated with a luciferase reporter assay focused on 3′ UTR (43). Similarly, sequence complementarity between miR-181c-5p seed sequence and Vegfa 5′UTR is limited, which supports an indirect regulation. Yet, the upstream target in this study remains unidentified. Although this miR-181c-5p/*Vegfa* -axis has not been reported in kidney earlier, endothelial modulation has been described in diabetic conditions, with contrasting effects across different tissues (19). In our study, we did not detect endothelial effects (such as renal capillary rarefaction) in mice treated with miR-181c-5p inhibitor.

Balanced expression of *Vegfa* is essential for renal physiology, where podocytes/pericytes are the exclusive source for its renal expression (44–46). Interestingly, clinical and preclinical data observed glomerular injury and renal TMA as a consequence of reduced renal *Vegfa* expression, for instance in patients treated with a Vegfa antibody (bevacizumab) and *Vegfa* knockout animal models (45, 47, 48). Patients treated with a less potent Vegfa antibody (ranibizumab) show less renal toxicity, which refers to dose-dependent effects (49). These observations strongly correspond with our histological findings in HF mice that died prematurely, and underpin the threefold higher predisposition for TMA development. The variable onset of TMA in this study likely associates with expression variation of *Vegfa*, model-dependent factors, and reflects the variable clinical onset (50). These findings support a recent study that emphasizes the regulatory role of miRNAs in different disease mechanisms of TMA (51).

**Figure 6:**
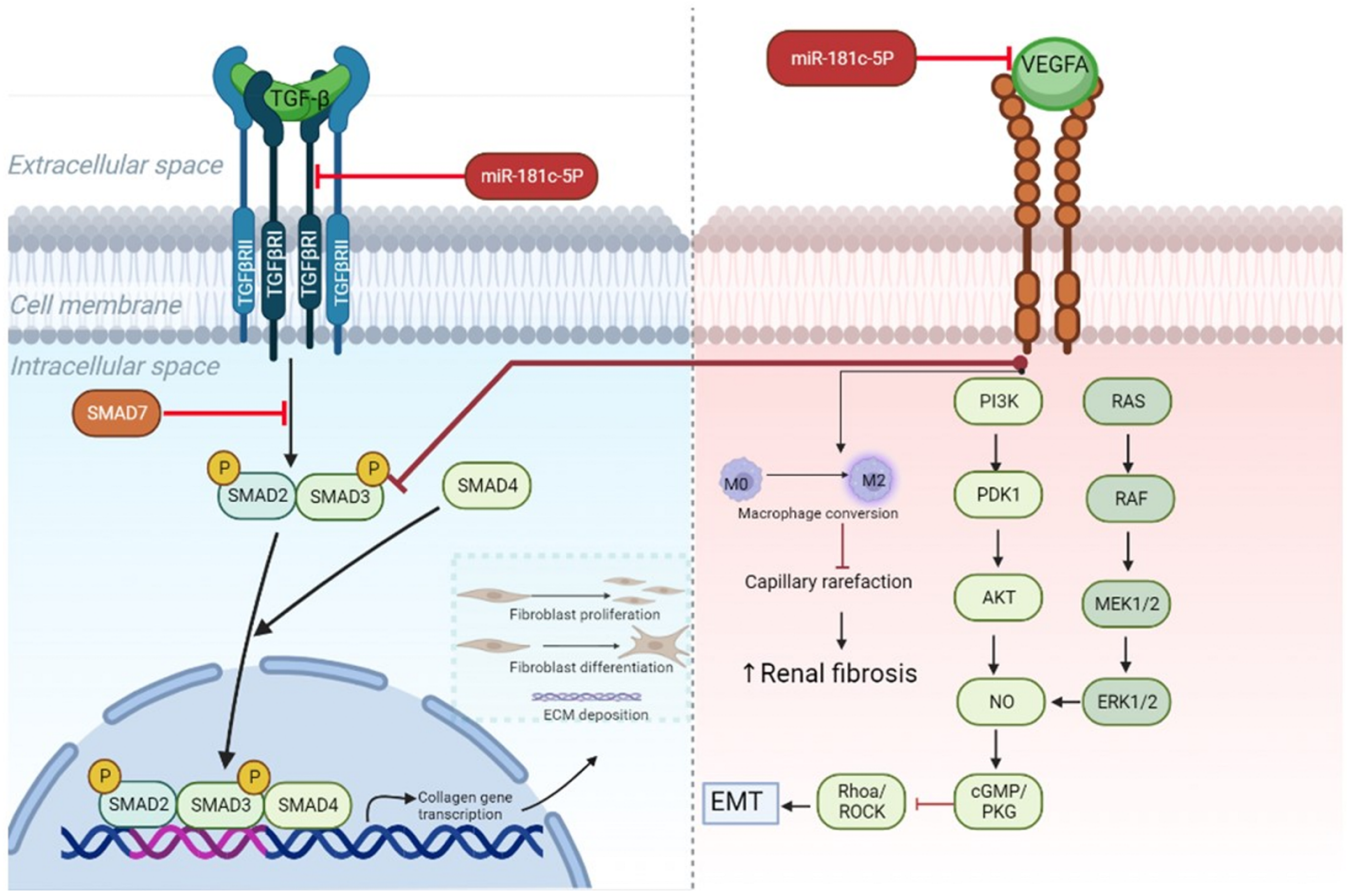
MiR-181c-5p-modulated pathways in heart and kidney. TGF-β downstream signaling induces activation of SMAD2/SMAD3 proteins and subsequent complex formation with SMAD4. By entering the nucleus, the protein complex can initiate transcription of genes involved in fibrosis, such as collagen, which after translation leads to extracellular matrix deposition, fibroblast proliferation, and differentiation from resting fibroblasts into myofibroblasts. All these processes drive fibrogenesis. In cardiac tissue miR-181c-5p inhibition reduces the levels of *Tgfbr1*— which is a type 1 receptor of TGF-β—and cooperates in this way to block myocardial fibrosis. Recent research demonstrates anti-fibrotic effects of Vegfa signaling in kidney (55). This can occur via direct targeting of TGF-β signaling by blocking SMAD3, while endothelial mesenchymal transition (EMT) is hampered through PI3K/AKT and ERK1/2 signaling activation. Furthermore, preventing capillary rarefaction by its proangiogenic properties, like conversion of resting M0 in activated M2 macrophages, hinders renal fibrosis. Inhibition of miR-181c-5p in renal tissue results in lowered expression levels of *Vegfa*, which reduces downstream signaling and could promote increased fibrotic activity. ECM, extracellular matrix; EMT, endothelial mesenchymal transition; Tgf-β, transforming growth factor β; Vegfa, vascular endothelial growth factor A; TgfbrI, transforming growth factor β receptor I.

In line with *Vegfa* knockdown studies, we identified very mild glomerular damage in healthy mice (52), but the extended areas of interstitial fibrosis were less common in literature. A few studies reported similar findings with increased levels of glomerulosclerosis and tubulointerstitial fibrosis after VEGF-receptor inhibition (53, 54). However, the underlying mechanism is unclear. Recent evidence points out to different direct and indirect pathways (Figure 6), while researchers also report the involvement of a pericyte-to-myofibroblast activation (54).

### 4.1 Limitations

This study must be interpreted in the light of the following limitations. Unforeseen death, particularly in HF + INH group, resulted in fewer data points and limited collection of plasma and urine samples, especially in the most severely affected animals. Therefore we lack clear functional data of the kidney in this group (Supplementary Figure S3), and the study must be assessed in the light of survivorship bias in the HF + INH group (only the least affected animals were analyzed in full). While we acknowledge that ideally both sexes should be examined, we only studied male mice due to practical considerations. Results obtained in mice might not fully translate to humans due to interspecies variations. Finally, this short-term study might not capture the long-term effects of miR-181c-5p modulation on HF progression, and diastolic dysfunction was only mildly affected in the mouse model on echocardiography (E/A and E/e’ did not reach statistical significance).

### 4.2 Conclusions and future perspectives

Briefly, this study provided new insights in the dual role of miR-181c-5p inhibition in heart and kidney and (1) identifies *Tgfbr1*-signaling as direct/indirect target of miR-181c-5p in the failing heart, (2) confirms that miR-181c-5p inhibition mildly attenuates HF progression by reducing cardiac fibrosis, which makes it an interesting target for fibrotic treatment, (3) establishes miR-181c-5p as key player for renal structure in healthy and HF conditions, (4) identifies renal *Vegfa*-signaling as target of miR-181c-5p, direct or indirect, and (5) emphasizes the additional value of multi-organ approach in systemic miRNA interventions to monitor comorbid organs.

Based on the promising therapeutic potential of miR-181c-5p inhibition for cardiovascular diseases as stated in literature, these findings are of considerate interest regarding the comorbid nature of cardiovascular pathology. Despite the beneficial effects in the heart, miR-181c-5p downregulation has detrimental effects on the kidney. Future research might reveal if cardiac-specific inhibition of miR-181c-5p could function as new anti-fibrotic target in the heart and could improve cardiac function to a larger extent when adverse effects from multi-organ crosstalk are eliminated. Further validation of *Vegfa* and *Tgfbr1* as organ-specific targets of miR-181c-5p in renal and cardiac tissue is required, but miR-181c-5p seems a valid target for further research on (cardio)-renal dysfunction and TMA.

## Data Availability

The original contributions presented in the study are included in the article/**Supplementary Materials**, further inquiries can be directed to the corresponding author.
