# Supplementary material for: Divergent cardiac and renal effects of miR-181c-5p inhibition in a rodent heart failure model
Source: Front Cardiovasc Med. 2024 Apr 25;11:1383046. doi: 10.3389/fcvm.2024.1383046 (PMC11079209; doi:10.3389/fcvm.2024.1383046)
Supplement: Supplementary file 1 [file Datasheet1.docx]

Supplementary Material

**
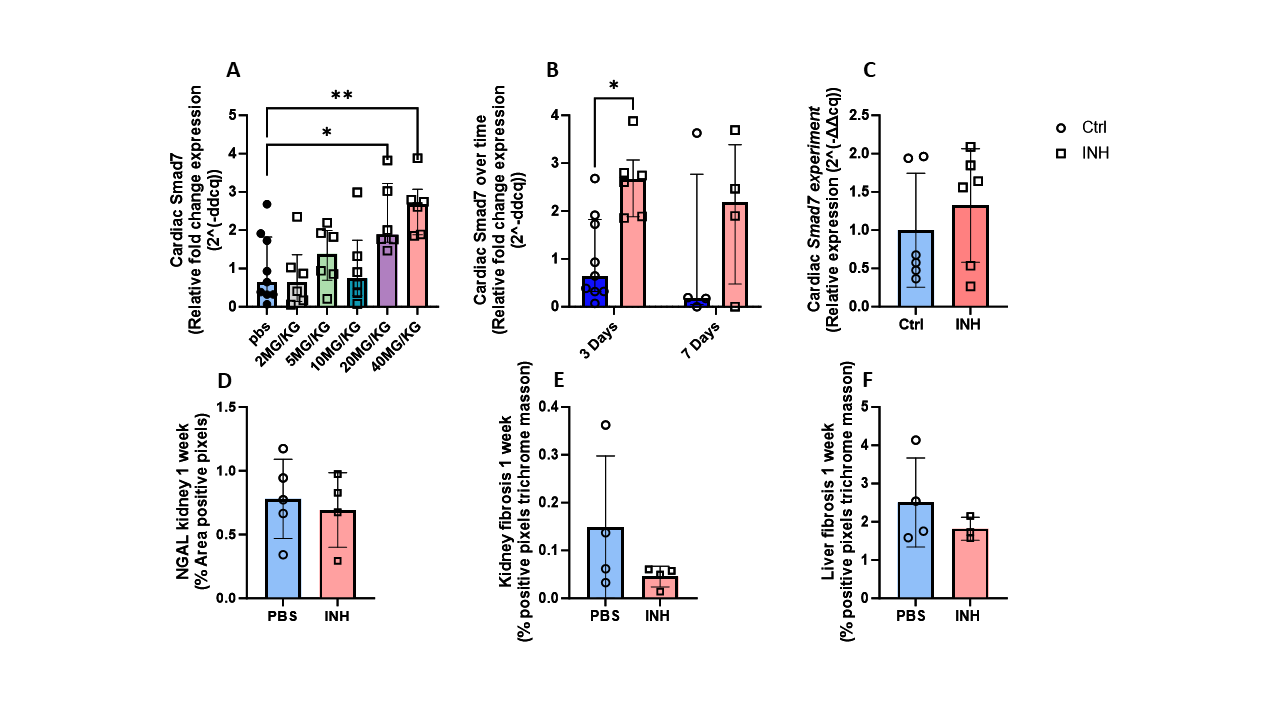
**

**Supplementary Figure 1.** Dose-ranging study and oligonucleotide-related toxicity. **(A)** Relative expression of *Smad7* in cardiac tissue three days after subcutaneous injection of miR-181c-5p antagomir at different doses (2,5,10,20,40 mg/kg) compared with PBS. A dose of 40mg/kg shows the highest targeting activity of *Smad7*. One-Way ANOVA with Dunnett’s multiple comparison correction with PBS as reference group ( N=6-9 per group). **(B)** Relative expression of *Smad7* in cardiac tissue at three days or one week after subcutaneous injection of either miR-181c-5p antagomir (dose 40mg/kg) or PBS. Average targeting activity remains high after 7 days, but with higher expression variation, likely associated with smaller group size. Two-Way ANOVA with Šidák’s multiple comparison test (N=4-9 per group). **(C)** Relative expression of *Smad7* in cardiac tissue during the final study: miR-181c-5p inhibitor at 40mg/kg after six weeks of weekly injections compared to scrambled antagomir. The graph demonstrates a similar, though less pronounced trend between miR-181c-5p and scrambled antagomir compared to graph B). The discrepancies might be associated with the considerable level of statistical variation in our qPCR results as well as a one day delay in the final experiment (N=6 per group). **(D)** Renal toxicity one week after subcutaneous injection of either miR-181c-5p antagomir or PBS determined with Neutrophil gelatinase-associated lipocalin (NGAL) staining. **(E)** Renal fibrosis one week after subcutaneous injection of either miR-181c-5p antagomir or PBS determined with Masson’s Trichrome staining. **(F)** Liver toxicity determined with Masson’s trichrome staining one week after injection of miR-181c-5p antagomir or PBS. H&E staining of liver did not show any abnormalities (data not shown). A-C) Relative expression is determined with RT-qPCR according to 2^(-delta delta cq) method; *p<0.05, **p<0.002.

**
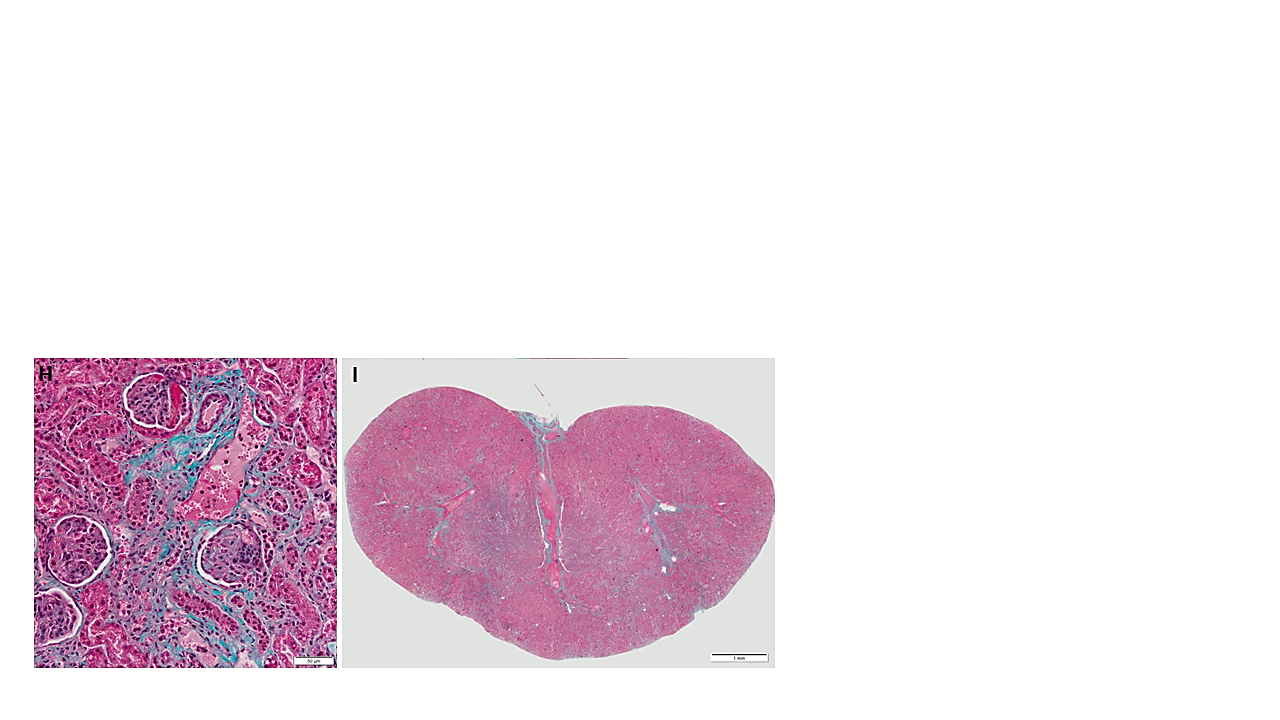
**


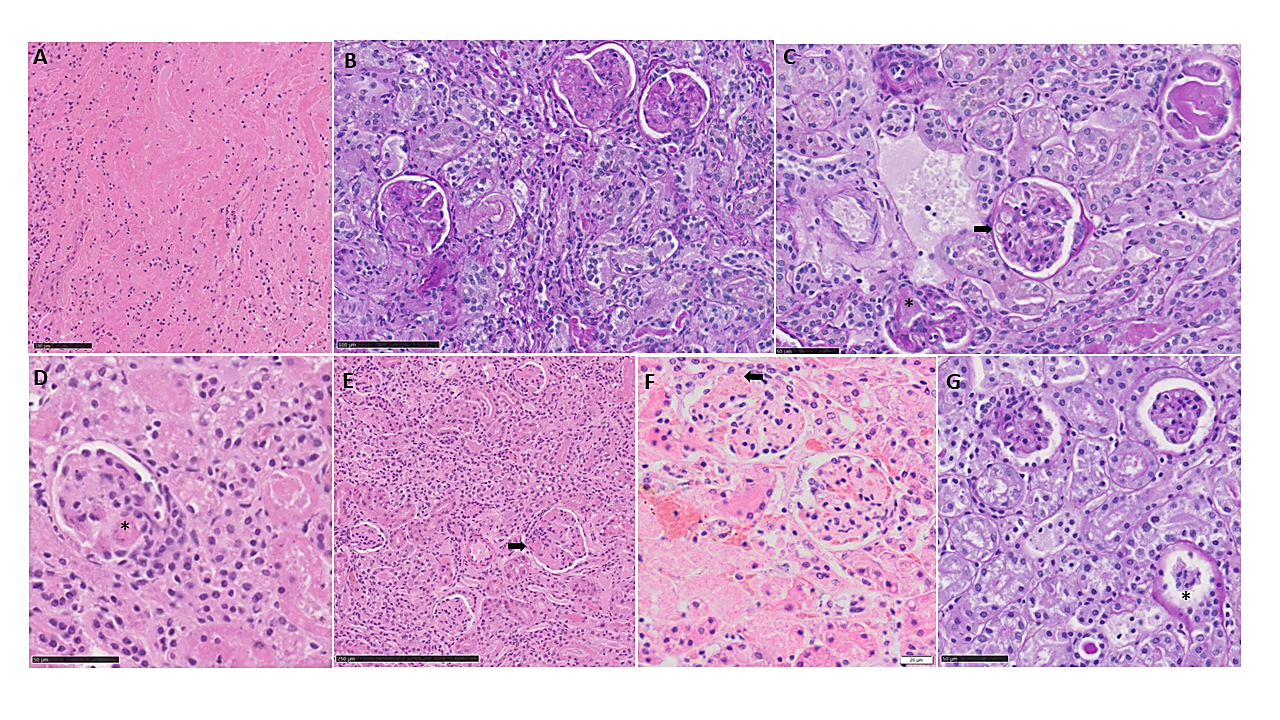


**Supplementary Figure 2.** Thrombotic microangiopahy (TMA) associated renal histology. **(A)** Tubular necrosis with inflammatory infiltration on H&E, **(B)** Bloodless aspect of glomeruli on PAS staining fitting with TMA phenotype, **(C)** Glomerulus presenting with fibrin clot (*), one with thickened capillary loops (arrow) and a degenerating glomerulus on PAS staining. **(D)** Glomerulus showing thrombus (*) while surrounded by necrotic tubules on H&E, **(E)** Hypertrophic glomerulus with thrombus (arrow) on H&E, **(F)** Glomerulus demonstrating fragmented red blood cells (arrow) while surrounded by necrotic tubules on H&E, **(G)** Atrophic glomerulus (*), **(H,I)** Renal fibrosis on Masson’s trichrome staining.


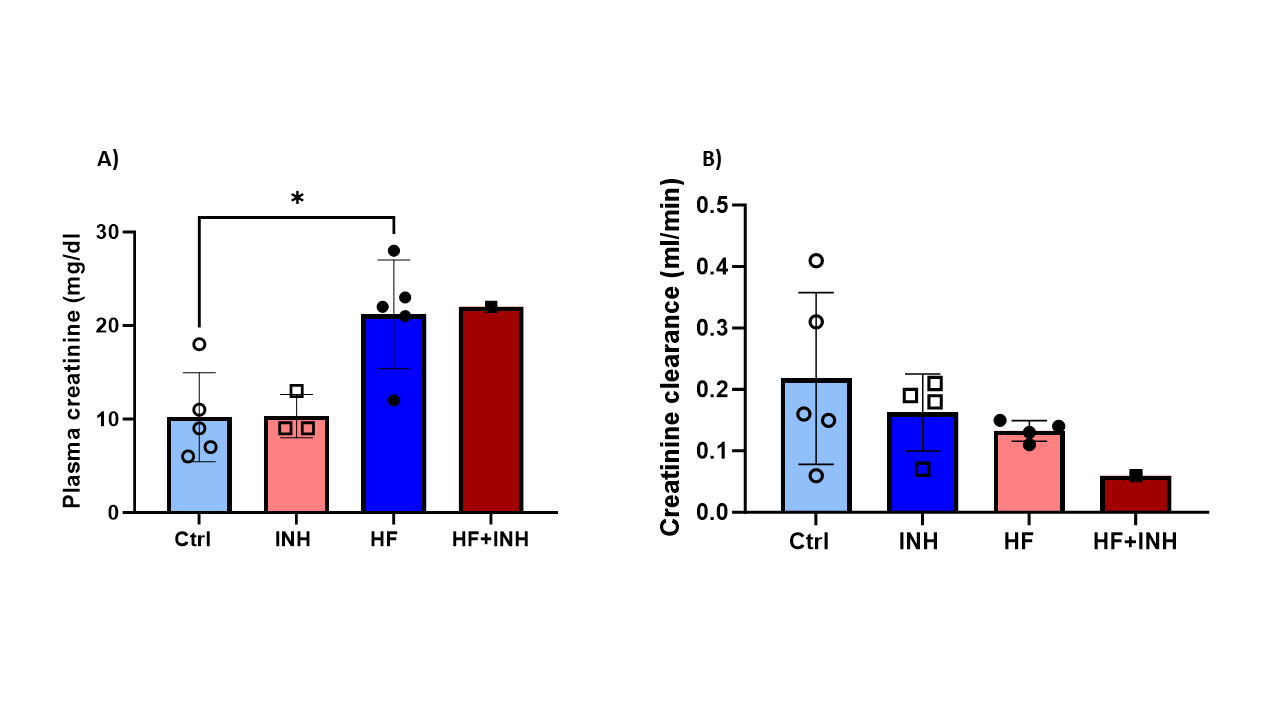


**Supplementary Figure 3**. Renal function determined with (A) Plasma creatinine, and (B) Creatinine clearance. Low sample size in HF+INH group is not suitable for statistical testing. N=1-5 per group, Two-Way ANOVA, Šidák test for multiple comparisons, *p<0.05.

Supplementary Table 1. Oligonucleotide sequences

| Oligonucleotide | Sequence |
| --- | --- |
| Mmu-miR-181c-5p antagomir | CCGACAGGTTGAATGT |
| Scrambled antagomir | ACGTCTATACGCCCA |

Supplementary Table 2. Target selection with prediction programs (miRSystem, mirDIP 4.1, in house gene set enrichment analysis), pathway analysis (DIANA tools, in house target analysis) and literature.

| *Hsa-miR-181c-5p* | Prediction programs | Pathway analysis | Literature |
| --- | --- | --- | --- |
| *TGFBR1*  *(Transforming Growth Factor ß Receptor 1)* | miRSystem: 6/7  mirDIP: high power  In house: p=0,0006 | DIANA tools: p=0,001 | *(1)* |
| *SMAD7*  *(SMAD Family Member 7)* | mirPath v3: CLIP  miRSystem: 7/7  mirDIP: very high power  In house: p=0,0006 | DIANA tools: p=0,008 | (2-4) |
| *VEGFA*  *(Vascular Endothelial Growth Factor A)* | In house: p=0,00004 | In house:P<0.001 | *(5, 6)* |

*CLIP= cross-linking immunoprecipitation; criteria for gene selection were described as follows: genes are either listed in 1) at least two prediction programs with adequate power (miRSystem: validated in ≥5 programs out of 7, mirDIP: ranked ≥ high power, DIANA mirPath: validation with CLIP, in house: p≤0,001), or 2) identified in both pathway analysis and at least one target prediction database, 3) and/or validated and described as target in a scientific article. Relevance for heart failure-related processes, like hypertrophy, fibrosis or endothelial function was a prerequisite for gene selection.*

Supplementary Table 3. Stability values and corresponding ranking (most to least stable) of potential reference genes for mRNA targets of miR-181c-5p in heart and kidney evaluated in different validation programs.

| Heart RNA | | | | |
| --- | --- | --- | --- | --- |
| *Reference gene* | **RPLPO** | **YWHAZ** | **GAPDH** | **ACTIN** |
| *geNorm M* | 0.50 | 0.65 | 1.58 | 0.86 |
| *NormFinder* | 0.33 | 0.88 | 2.20 | 0.84 |
| *RefFinder* | 1.19 | 2.06 | 4 | 2.06 |
| *Stability Ranking* | **1** | **2** | **4** | **3** |
| Kidney RNA | | | | |
| *geNorm M* | 1.15 | 1.65 | 1.1 | 1.22 |
| *NormFinder* | 13.53 | 13.57 | 8.29 | 1.51 |
| *RefFinder* | 3 | 4 | 1.68 | 1 |
| *Stability Ranking* | **3** | **4** | **2** | **1** |

Supplementary Table 4. Stability values and corresponding ranking (most to least stable) of potential reference genes for miR-181c-5p expression in heart and kidney evaluated in different validation programs.

| Heart miRNA | | | | |
| --- | --- | --- | --- | --- |
| Reference gene | **SNO142** | **SNO202** | **SNO234** | **RNU6B** |
| geNorm M | 1.37 | 1.37 | 4.86 | 7.02 |
| NormFinder | 3.15 | 0.69 | 6.18 | 8.38 |
| RefFinder | 1.86 | 1.19 | 3.22 | 2.83 |
| Stability Ranking | **2** | **1** | **3** | **4** |
| Kidney miRNA | | | | |
| geNorm M | 1.19 | 1.8 | 1.3 | 1.02 |
| NormFinder | 10.46 | 10.53 | 12.18 | 12.87 |
| RefFinder | 1.86 | 1.57 | 2.45 | 2.83 |
| Stability Ranking | **1** | **2** | **4** | **3** |

**References**

1. He X, Liu Z, Peng Y, Yu C. MicroRNA-181c inhibits glioblastoma cell invasion, migration and mesenchymal transition by targeting TGF-β pathway. Biochem Biophys Res Commun. 2016;469(4):1041-8.

2. Li Y, Wang H, Li J, Yue W. MiR-181c modulates the proliferation, migration, and invasion of neuroblastoma cells by targeting Smad7. Acta Biochim Biophys Sin (Shanghai). 2014;46(1):48-55.

3. Fu Y, Tang Y, Wang J, Guo Z. MicroRNA-181c Suppresses the Biological Progression of Osteosarcoma via Targeting SMAD7 and Regulating Transforming Growth Factor-β (TGF-β) Signaling Pathway. Med Sci Monit. 2019;25:4801-10.

4. Jankauskas SS, Mone P, Avvisato R, Varzideh F, De Gennaro S, Salemme L, et al. miR-181c targets Parkin and SMAD7 in human cardiac fibroblasts: Validation of differential microRNA expression in patients with diabetes and heart failure with preserved ejection fraction. Mech Ageing Dev. 2023;212:111818.

5. Solly EL, Psaltis PJ, Bursill CA, Tan JTM. The Role of miR-181c in Mechanisms of Diabetes-Impaired Angiogenesis: An Emerging Therapeutic Target for Diabetic Vascular Complications. Front Pharmacol. 2021;12.

6. Hourigan ST, Solly EL, Nankivell VA, Ridiandries A, Weimann BM, Henriquez R, et al. The regulation of miRNAs by reconstituted high-density lipoproteins in diabetes-impaired angiogenesis. Sci Rep. 2018;8(1):13596.
